# Undergraduate Public Health, Lessons Learned from Undergraduate Health Administration Education

**DOI:** 10.3389/fpubh.2015.00038

**Published:** 2015-02-26

**Authors:** Joel M. Lee, Leonard H. Friedman

**Affiliations:** ^1^John A. Drew Professor of Healthcare Administration, College of Public Health, University of Georgia, Athens, GA, USA; ^2^Milken Institute School of Public Health, The George Washington University, Washington, DC, USA

**Keywords:** undergraduate students, public health, education, medical, undergraduate public health education, lessons learned, MPH, MHA

## Introduction

The rapid growth in the number of undergraduate programs in public health reflects a similar earlier experience in health administration education in North America and offers a set of experiences that we may both learn from and contribute to. In a similar fashion to the public health discipline first awarding the MPH degree, the entry level health administration credential began as a masters degree in 1933, and subsequent degrees followed awarding the Master of Health Administration (MHA) and similar degrees. In the 1970s, undergraduate programs in health administration began to proliferate, and the graduate programs were “initially very cautious about establishing formal relationships” (1) requiring consideration of a set of questions about the relationship between baccalaureate and masters degree education in the discipline. This experience may be beneficial in addressing the more recent undergraduate/graduate degree relationship in public health. While there are numerous issues to consider, this commentary will address several of the most important issues. In 1975, Andrew Patullo, then Senior Vice-President of the W. K. Kellogg Foundation noted that “in light” of health administration being associated with graduate education, the development of undergraduate education presented a perplexing development, and nonetheless, the establishment of baccalaureate programs was seen as a “logical sequential development” (2) Table 1 presents a series of key events in the evolution of baccalaureate education in health administration, and a surprisingly similar set of parallel events in the more recent evolution of public health education. While it is not a certainty that parallel events will continue, the authors believe that anticipating subsequent issues from health administration will facilitate the maturation of undergraduate public health education.

**Table 1:** **Comparable events in the development of undergraduate health administration and public health education**.

Undergraduate health administration education	Undergraduate public health education
Undergraduate Programs in public health were offered as early as the 1920s (1)	Undergraduate programs in public health began in the 1970s (3)
1980, establishment of the AUPHA Undergraduate Task Force (2)	2007, establishment of ASPH Task Force on undergraduate public health education (4)
In 1970, AUPHA found that more than 100 Colleges and Universities in the U.S. and Canada were offering “some kind of work in the field of health administration at the baccalaureate level” (5)	A 2008 survey by the AAC&U identified 137 members with public health majors, minors, or concentrations (6)
1975, The Commission on Education for Health Administration recommends health administration education be offered in a variety of settings with diverse educational strategies including undergraduate programs (5)	2003, The Committee on Educating Public Health Professionals for the twenty-first century recommends that all undergraduates should have access to education in public health (7)
	2012, ASPH *Recommended Critical Component Elements of an Undergraduate Major in Public Health* Full Educational Model and Report (13)
1975, Undergraduate Education for Health Services Administration: Proceedings of the First Undergraduate Faculty Institute (2).	2006, Consensus Conference on Undergraduate Public Health Education, sponsored by the Association for Prevention Teaching and Research, the Association of Schools of Public Health (ASPH), and the Council of Colleges of Arts and Sciences (CCAS).(8)
1980, Establishment of the AUPHA Certification, Undergraduate Review Committee (8)	2014, Establishment of CEPH Standalone Baccalaureate Program Accreditation (9)
1985, First published report: *Baccalaureate Health Administration Education: Curriculum Models and Issues* (8)	2007, First published report: *The Educated Citizen and Public Health: A Consensus Report on Public Health and Undergraduate Education* (10)

## Pathways for Graduates

As noted in our companion papers (11, 12), undergraduate programs in public health have been described as a pathway for several alternatives, liberal education for an informed citizenry, undergraduate education for professional programs including law or medicine, preparation for masters degrees in the discipline, and preparation for entry level positions in the profession. In health administration education, programs typically followed the same pattern and many students selected the opportunities for entry level and frequently midlevel career positions such as hospital department heads and associate administrators. These opportunities were enhanced by the fact that many recent masters level graduates had no more work experience than their baccalaureate graduate counterparts, had higher target incomes, and were less interested in positions in rural and underserved areas. Similar opportunities may well exist for the baccalaureate public health graduate. However, as MHA programs proliferated, their graduates have displaced many baccalaureate graduates for these opportunities and the MHA has frequently become the entry level credential repositioning baccalaureate graduates away positions that they previously pursued. This degree escalation has resulted in many health administration baccalaureate graduates returning to graduate education for credentials that they may not have planned to pursue in order to obtain positions they desire. In a similar fashion, concurrent to the growth in undergraduate public health education, there has also been rapid growth in graduate public health education. A similar phenomenon may affect public health.

## Introductory Coursework at Both Degree Levels

There is a common and somewhat unique characteristic to education in each of the two disciplines as baccalaureate education was preceded by entry level graduate education and curriculum was then “reverse engineered” from the established graduate education that preceded it. This contrast, with many other professions where completing an undergraduate curriculum, is a prerequisite to graduate education in the discipline. For example, a Bachelor of Science in Nursing (BSN) degree is almost a universal expectation of applicants to a Master of Science in Nursing (MSN) program. While an undergraduate business degree is not a universal requirement for Master of Business Administration (MBA) programs, many programs have some undergraduate introductory course prerequisite requirements in areas such as accounting and economics. As there are rarely prerequisite areas or specific degree requirements in both graduate health administration and public health, introductory courses in disciplinary content are a norm in both MHA and MPH degree curricula.

This is noteworthy as introductory disciplinary coursework is a most unusual component in graduate curricula. As undergraduate programs in public health proliferate, graduate admissions are likely to include a mix of students with undergraduate introductory public health course content and degrees, and those with other undergraduate majors without the relevant undergraduate introductory coursework. In the absence of MPH programs requiring undergraduate coursework or degrees, it will be impossible to remove these introductory courses from graduate education. The mix of backgrounds of matriculating MPH students creates a complication for the graduates of baccalaureate programs, and curriculum design for the graduate programs. Waiving or substituting these courses for baccalaureate graduates presents an interesting question for the graduate programs in assessing the difference between an undergraduate “introduction to ….” course and a graduate “introduction to ….” course. In some cases noting this, undergraduate health administration students choose a different graduate degree such as business or public administration. These degrees frequently led the baccalaureate health administration graduates to careers in other disciplines such as banking or government and excellent people were lost to health administration careers.

In other cases as undergraduate health administration faculty, the authors have experienced calls from undergraduate students in health administration expressing concern for their choice in pursuing an MHA degree as they were using the same textbooks in courses with similar titles, and earning high test grades without studying. While this typically resolved itself as students move beyond the introductory courses, the overlap does present issues related to best use of time and money for the health administration baccalaureate graduate as well as the public health undergraduate.

## Duplication of Curriculum Content

Frequently, faculty teaching at both the undergraduate and graduate levels in health administration speaks to the importance of considering variations in depth, breadth, and competencies to differentiate courses at the two levels, and the need to carefully consider alternatives if a graduate course were to be waived. Faculty in public health needs to be aware of this issue and carefully consider it in course and curriculum design as subsequent coursework will rely on introductory course competency. Experience in health administration education also demonstrates that undergraduate advisors must play a careful role in addressing this issue for their students considering graduate education and selecting appropriate programs in the same discipline. Undergraduate advisors in public heath need to be aware of these issues in counseling their students.

## Program Location and Faculty

Achieving excellence is also an issue that is shared by the two disciplines. While some undergraduate programs are co-located in the same unit as a graduate program with faculty teaching at both levels, some undergraduate programs are located in other disciplinary units. In the case of health administration, this included allied health, political science, nursing, business, or the humanities. Health administration faculty in some of these settings previously focused on teaching other subjects. In some instances, the faculties were educated in other disciplines retraining themselves to teach health administration courses. Public health programs share similar units and similar issues based on rising interest in public health and in some cases declining interest in other majors.

Young health administration programs typically had small faculties, in some cases, as few as two instructors each offering eight diverse course preparations in an academic year. Teaching was complemented by substantial advising responsibilities and the establishment and supervision of student field placements and career planning. This also appears to be the case in public health with programs in a variety of academic units and recent demand resulting in summer short courses to prepare existing faculty to teach public health courses. As health administration has matured, faculty credentials better reflect curriculum content, and the same may be the case in public health. However, the role of disciplinary research and teaching must also be considered for faculty engaged in large and diverse teaching responsibilities.

## Class Size

An additional consideration in health administration is class size. Undergraduate students are searching for careers and frequently change majors based upon something that excites them. In some years, the health administration discipline has been extremely popular based upon awareness initiated by a public event or the media, resulting in very high demand by students for the major. The same may be true in public health based upon world events or a popular movie with a public health focus. In some health administration, programs enrollment is open and not capped in size. However, in others, based upon limits on capacity and/or efforts to select the best students, there are formal requirements for admission including application, prerequisite coursework, minimum grade point averages, work experience, and interviews. The same issue may apply to undergraduate public health. For example, the current attention to Ebola may increase interest and demand for undergraduate degrees and exceed program enrollment capacity.

## Academic Program Credentialing and Association Membership

In health administration, the Association of University Programs in Health Administration (AUPHA) was established as an organization of graduate programs. Following the establishment of a critical mass of undergraduate programs, AUPHA needed to consider its relationship with the undergraduate programs. In a similar fashion, the accrediting body, the Council on Accreditation in Health Management Education (CAHME) needed to evaluate its role in undergraduate education. Following initial resistance, AUPHA revised its bylaws to permit undergraduate programs full membership in the association including seats on its governing board, and many undergraduate faculty have served as board chair. This was at least in part based on a decision that a single organization would be preferable to the undergraduate programs creating a second organization. External program review is important to quality improvement. For 25 years, AUPHA and CAHME have chosen “Undergraduate Program Certification” as the undergraduate program credentialing mechanism through an AUPHA panel review process as an alternative to “accreditation.” This on-site panel review takes place at AUPHA’s annual meetings as an alternative to CAHME site visits offering a more financially affordable and inclusive strategy.

In a similar fashion, the Association of Schools and Programs in Public Health (ASPPH) and the Council on Education in Public Health (CEPH) represent public health education. ASPPH will potentially need to consider its relationship with undergraduate programs as they proliferate or potentially risk the development of a parallel undergraduate program association. In 2013, CEPH approved offering “Standalone Baccalaureate Program” accreditation, which may be more exclusive due to the high cost of site visits. It will be interesting to watch the evaluation of these two credentialing processes and the number of participants as they evolve.

## Credentialing of Graduates

In the area of personal credentialing of professionals each discipline has alternative professional associations and credentialing bodies. The American College of Healthcare Executives (ACHE) is the leading professional affiliation for students and graduates in health administration practice. At a point in the past, ACHE affiliation required an MHA for membership, this requirement has now been removed and baccalaureate graduates are eligible for affiliation. ACHE also offers examination-based board certification in healthcare management; however, a graduate degree is required for the examination. In public health, the American Public Health Association (APHA) is the largest membership association and has no academic requirement for membership. The National Board of Public Health Examiners (NBPHE) was established in 2005 as the public health professional certification body. Although requests have been made in regard to baccalaureate eligibility, eligibility for the examination presently requires a graduate degree from a CEPH-accredited school or program to qualify for certification.

## Conclusion

Each of these considerations contributes to the educational enterprise as well as definition of the health administration and public health professions. Health administration and public health education share a variety of issues as they are atypically disciplines where graduate education preceded undergraduate education. This narrative is an opportunity to demonstrate that the recent growth of undergraduate public health education is not unique. While health administration may not offer all of the answers to integration of the public health baccalaureate education into a discipline with established graduate degree entry level profession, we may not only learn from other disciplines but also contribute to their evolution.

## Conflict of Interest Statement

The authors declare that the research was conducted in the absence of any commercial or financial relationships that could be construed as a potential conflict of interest.
